# Utilization of methylene blue–based submucosal injection to identify residual neuroendocrine tumor in the colon after endoscopic biopsy sampling

**DOI:** 10.1016/j.igie.2023.01.009

**Published:** 2023-02-01

**Authors:** Weina Jing, Yuxiang Chen, Kai Deng

**Affiliations:** 1 Department of Gastroenterology & Hepatology, West China Hospital, Sichuan University, Chengdu, Sichuan, China; 2 Sichuan University, Oxford University Huaxi Gastrointestinal Cancer Centre, Department of Gastroenterology & Hepatology, West China Hospital, Sichuan University, Chengdu, Sichuan, China

## Abstract

A woman underwent colonoscopy with biopsy sampling that revealed a tiny neuroendocrine tumor (NET) about 5 cm from the anus. After several months, the biopsy sampling site healed completely, making the identification of the residual NET difficult. The specific location of the lesion could not be confirmed even under magnifying endoscopy and EUS. When a higher concentration of methylene blue was injected into the submucosa, the residual lesion was exposed. The endoscopist then inferred the likely location of the lesion on magnifying endoscopy on the basis of the marker and the location of the lesion after injection of methylene blue. Finally, the residual lesion was successfully removed, and the postoperative pathology confirmed that the resected lesion was a residual NET measuring 1.5 × .5 mm.

A 69-year-old woman underwent colonoscopy with biopsy sampling that revealed a small neuroendocrine tumor (NET) G1 about 5 cm from the anus. After a few months, the patient decided to undergo endoscopic resection to remove the lesion. However, the biopsy sampling wound healed completely, and the residual lesion was extremely tiny, making the identification of the residual NET more difficult even with magnifying endoscopy and EUS (Fig. 1A and B).

**Figure 1:**
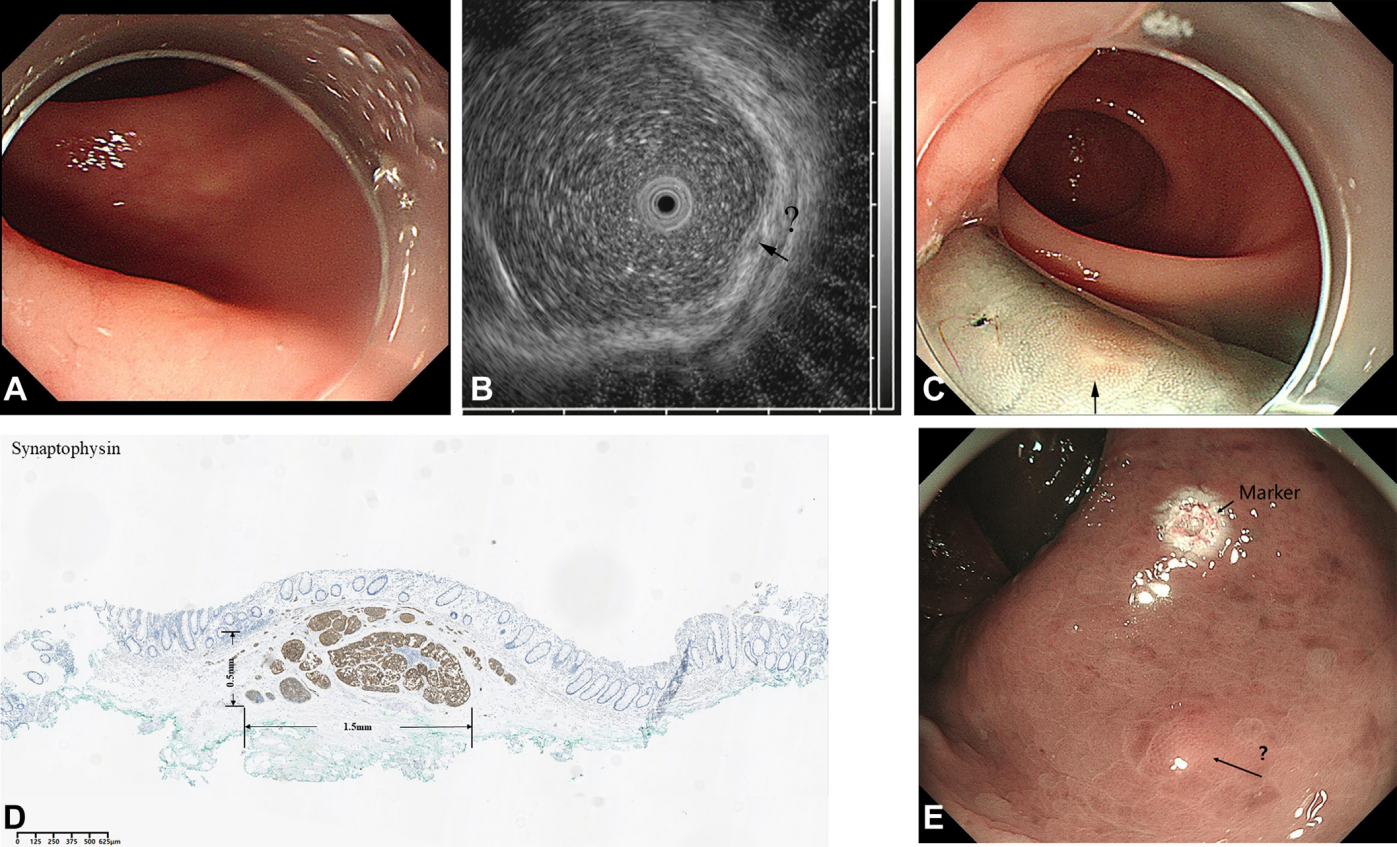
The tiny lesion of residual neuroendocrine tumor (NET) under endoscopy and pathology. **A,** No obvious manifestation for the residual lesion or biopsy sampling scar was observed during ordinary endoscopy. **B,** An indeterminate hypoechoic lesion in the lamina propria of the mucosa was found 5 cm from the anus (*black arrow*). **C,** After injection of methylene blue, a yellowish smooth lesion was noted (*black arrow*). **D,** Pathologic findings indicated the lesion was a NET 1.5 × .5 mm in size (as measured by a pathologic section scale). **E,** The possible location of the residual lesion under magnifying endoscopy (*black arrow*).

Because the patient decided to undergo endoscopic resection of the lesion and injection of methylene blue is a routine procedure in endoscopic resection, the endoscopist attempted to inject methylene blue into the suspicious lesion to identify it. Surprisingly, when a colored solution of methylene blue (diluted it to approximately .2% using normal saline solution) was injected into the submucosa of the rectum, the residual lesion was exposed. It appeared as a flat bulge, 5 × 5 mm in size, with a smooth surface, and a relatively yellowish color (Fig. 1C). Subsequently, the endoscopist performed EMR to remove the lesion in its entirety. Finally, the postoperative pathology confirmed that the resected lesion was a residual NET measuring 1.5 × .5 mm (Fig. 1D). In addition, the endoscopist reviewed the entire examination process after the operation and judged the possible location of the lesion under magnifying endoscopy before injection of methylene blue according to the marker made under magnifying endoscopy and the location of the lesion after the injection of methylene blue (Fig. 1E, *arrow*). Through the above process (Fig. 2), we found that tiny lesions hidden in the lamina propria, such as small residual lesions after biopsy sampling, could be easily visualized by injecting a colored solution into the submucosa for enhancing the color contrast between the lesion and background mucosa.

**Figure 2:**
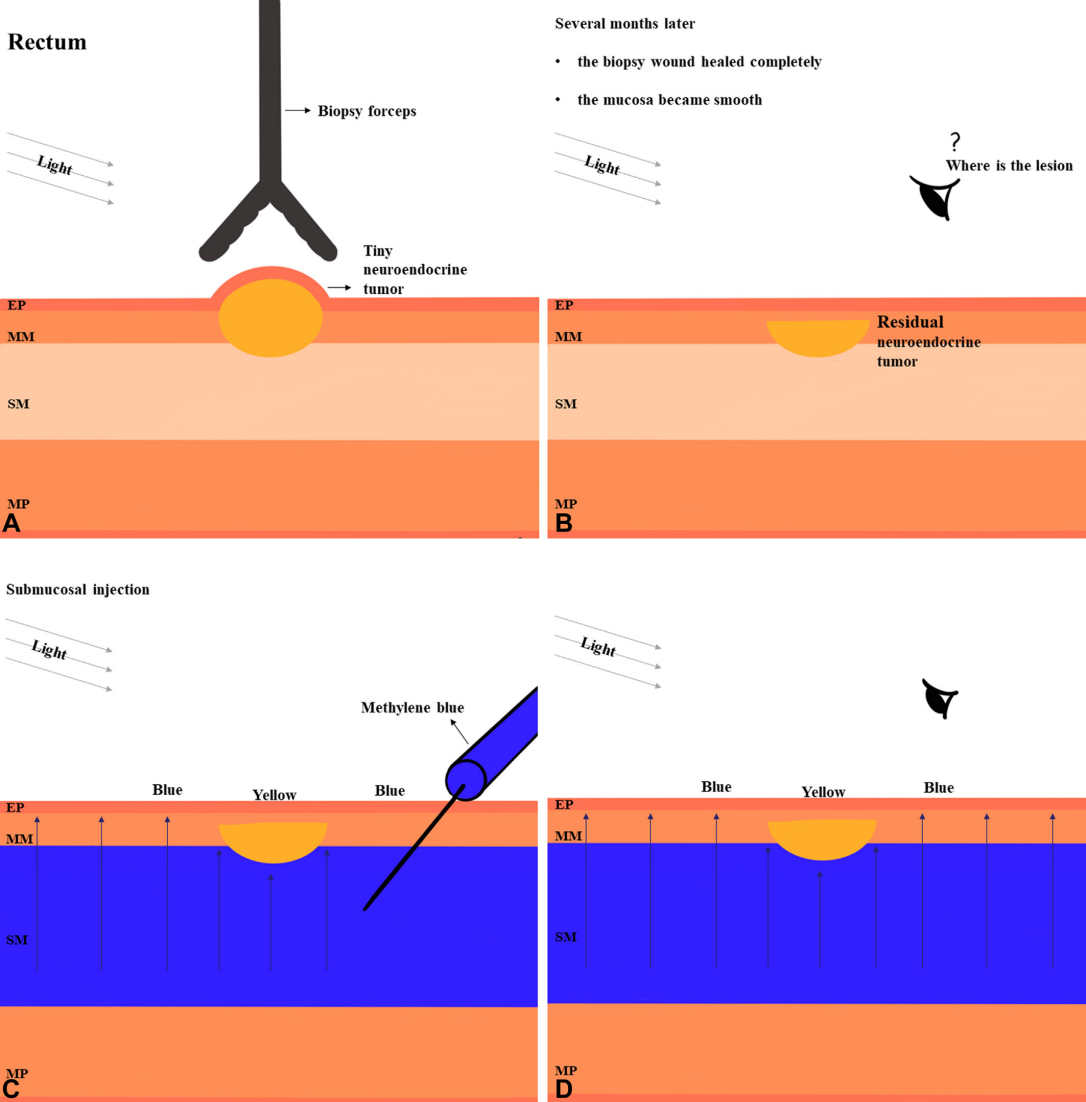
The procedure flowchart of injecting methylene blue to identify a tiny lesion of the residual neuroendocrine tumor (NET). **A,** A tiny NET was revealed by the colonoscopy with biopsy sampling. **B,** After several months, the biopsy sampling wound healed completely, and the mucosa was smooth. **C,** Injecting a higher concentration of methylene blue into the submucosa exposed the residual lesion. **D,** The residual lesion could be identified by increasing the color contrast between the lesion and background mucosa. *EP*, Epithelium; *MM*, muscularis mucosa; *SM*, submucosa; *MP*, muscularis propria.

